# In vivo imaging of cathepsin B in activated glia in the brain after orofacial formalin test

**DOI:** 10.1038/s41598-024-52854-2

**Published:** 2024-02-24

**Authors:** Sabrina L. McIlwrath, Amanda C. Carroll-Portillo, Henry C. Lin, Karin N. Westlund

**Affiliations:** 1 New Mexico VA Health Care System, Albuquerque, NM 87108 USA; 2 Division of Gastroenterology and Hepatology, University of New Mexico, Albuquerque, NM 87131 USA; 3 Department of Anesthesiology and Critical Care Medicine, University of New Mexico Health Sciences Center, Albuquerque, NM 87106 USA

**Keywords:** Fluorescence imaging, Glial biology, Pain

## Abstract

PURPOSE Cathepsin B (Cat B) is a cysteine lysosomal protease that is upregulated in many inflammatory diseases and widely expressed in the brain. Here, we used a Cat B activatable near-infrared (NIR) imaging probe to measure glial activation in vivo in the formalin test, a standard orofacial inflammatory pain model. The probe’s efficacy was quantified with immunohistochemical analysis of the somatosensory cortex. PROCEDURES Three different concentrations of Cat B imaging probe (30, 50, 100 pmol/200 g bodyweight) were injected intracisternally into the foramen magnum of rats under anesthesia. Four hours later formalin (1.5%, 50 μl) was injected into the upper lip and the animal’s behaviors recorded for 45 min. Subsequently, animals were repeatedly scanned using the IVIS Spectrum (8, 10, and 28 h post imaging probe injection) to measure extracellular Cat B activity. Aldehyde fixed brain sections were immunostained with antibodies against microglial marker Iba1 or astrocytic GFAP and detected with fluorescently labeled secondary antibodies to quantify co-localization with the fluorescent probe. RESULTS The Cat B imaging probe only slightly altered the formalin test results. Nocifensive behavior was only reduced in phase 1 in the 100 pmol group. In vivo measured fluorescence efficiency was highest in the 100 pmol group 28 h post imaging probe injection. *Post-mortem* immunohistochemical analysis of the somatosensory cortex detected the greatest amount of NIR fluorescence localized on microglia and astrocytes in the 100 pmol imaging probe group. Sensory neuron neuropeptide and cell injury marker expression in ipsilateral trigeminal ganglia was not altered by the presence of fluorescent probe. CONCLUSIONS These data demonstrate a concentration- and time-dependent visualization of extracellular Cat B in activated glia in the formalin test using a NIR imaging probe. Intracisternal injections are well suited for extracellular CNS proteinase detection in conditions when the blood–brain barrier is intact.

## Introduction

In response to peripheral tissue injury and/or inflammation, glial cells are activated in the central nervous system (CNS), brain and spinal cord. Their pro-inflammatory state is central in the development of chronic pain^1–7^. The study of pathological pain development has included  focus on microglia, innate immune cells in the CNS^8–10^. In the healthy, uninjured state, ramified microglia maintain tissue homeostasis by monitoring the CNS environment, clearing toxic substances and cells by phagocytosis, and facilitating synaptic connectivity^11^. Upon peripheral inflammation, microglia proliferate, and, along with invading macrophages, adapt an M1 pro-inflammatory state. They display distinct morphological and protein expression changes; many proteins such as microglia/macrophage-specific marker Iba1 significantly increase^12,13^. Activated microglia release pro-inflammatory mediators, cytokines, cyclic nucleotides, and neurotrophic factors that cause secondary activation of neighboring cells such as astrocytes^3,5,14,15^. In response, astrocytes alter their protein expression including increases in the expression of glial fibrillary acidic protein (GFAP)^1,16^. Autocrine effects of cytokine and cyclic nucleotide release on microglia include alteration of the subcellular location of peptidases, such as cathepsin, in support of immune functions^17,18^. Peptidases, such as cathepsin B (Cat B), have been identified as important participants in microglia/astrocyte mediated inflammatory central sensitization and neurodegeneration^7,15,19^.

Cat B is one of 11 cysteine lysosomal proteases and under normal conditions is found in endo-/lysosomal organelles. It functions as both an endo- and exopeptidase^20^. It is primarily involved in protein degradation and maintenance of cellular homeostasis^7,18,21,22^. Upregulation of Cat B is associated with many inflammatory diseases such as arthritis, pancreatitis, cardiovascular diseases, and cancer^23,24^. Cat B is expressed by activated microglia with intracellular Cat B participating in the expression and release of pro-inflammatory cytokines and neurotoxin^25–27^. In response to reactive oxygen species Cat B is also secreted and can modulate cell adhesion and receptor molecules as well as the extracellular matrix^28^. While intracellular molecules are extremely difficult to detect with an injectable probe, extracellular Cat B can be visualized using a compound that consists of a substrate molecule specific for this peptidase conjugated with a near infrared dye which fluoresces only upon cleavage^29,30^. Each protease-activatable fluorescent imaging compound molecule consists of two NIR dye molecules connected by linkers to either end of the protease-specific peptide chain spacer (exact sequence is proprietary information of PerkinElmer). The fluorescence is quenched due to the close proximity of the dye molecules. Upon cleavage by Cat B, the NIRF dye fluoresces^31^. A previous study used a protease-activatable near-infrared fluorescence (NIRF) imaging agent (“Cat B 680 FAST”) to visualize blood–brain barrier breakdown and CNS immune cell infiltration in a mouse model of multiple sclerosis^32^. Here we investigated if a similar activatable NIRF compound that fluoresces at even longer wavelengths (“Cat B 750 FAST”) could be used to visualize activated glia in the brain in vivo after inducing the formalin test, a rodent model of persistent inflammatory pain^33,34^.

Used for over 40 years, the formalin test is a standard inflammatory pain test. It has been extensively used to test analgesic properties of novel compounds and study the function of newly identified proteins in nociception^35,36^. Subcutaneous injection of a small volume of a 1–5% formalin solution produces a biphasic nocifensive response in rodents. Acute phase 1 behavior is thought to be driven by activation of nociceptors while the delayed phase 2 behavior has been attributed to central sensitization^12,33,37^. Persistent inflammatory orofacial pain models activate microglia and astrocytes in the CNS^38^. We used an in vivo imaging system (IVIS) to determine the efficacy of the Cat B activatable NIRF imaging probe injected directly into the cerebrospinal fluid (CSF) at the foramen magnum for in vivo measurement of brain glial activation by the orofacial formalin test. Immunohistochemistry was used to evaluate activation of this compound on glial cells and to ascertain if the probe would alter known protein expression changes.

## Methods

### Animals

All animal procedures were approved by the New Mexico Veteran’s Administration Health Care System Animal Component of Research Protocol (ACORP 20-A322) and performed in accordance with the National Institute of Health Guide for the Care and Use of Laboratory Animals (NIH Publications No. 80–23) revised 1996. A total of 12 adult male Sprague–Dawley rats (125–150 g, Envigo, Indianapolis, IN, USA) were used for this study. Animals were housed on a 12/12 reverse light cycle allowing testing during their active period. The rats were fed a soy protein-free diet (Teklad #2920x) ad libitum.

### Intracisternal injection of Cat B activable NIRF in vivo imaging probe

Animals were anesthetized with isoflurane (induction: 5% isoflurane in 0.8 L O_2_; maintenance: 3.5% isoflurane in 0.8 L O_2_), head and neck shaved, and the skin in the nape of the neck cleaned with betadine, followed by 70% ethanol. Animals were positioned nose down with the superior aspect of the neck hyperextended to palpate the depression of the cisterna cerebellomedullaris between the occipital bone of the skull and the atlas^39^. A 26-gauge, 5/8-inch long single-use needle attached to a 0.3 ml insulin syringe was inserted along the dorsal midline into the cisterna magna. A volume of 50 μl of the imaging probe (30, 50 or 100 pmol “Cat B 750 FAST”, PerkinElmer, Waltham, MA, USA) diluted in sterile phosphate-buffered saline (PBS) following manufacturer’s instructions was slowly injected. The needle was held in place for an additional 30 s to minimize cerebral spinal fluid and Cat B imaging probe leakage while breathing and heart rate were closely monitored^40^. The needle was then carefully removed, and the animal allowed to recover in its home cage.

### Orofacial formalin test

Four hours after Cat B imaging probe injection, the orofacial formalin test was induced. Animals were anesthetized with isoflurane (5% isoflurane in 0.8 L O_2_), and 1.5% formalin (50 μl diluted in 0.9% sterile saline; Sigma) was injected subcutaneously into the right upper lip using a 27-gauge needle^34^. Animals were transferred into a clean cage without bedding and video recorded for the following 40 min for offline quantification of nocifensive behavior, i.e. wiping and scratching of the perinasal area. At the end of the observation period, animals were returned to their home cages.

### IVIS Illumina optical imaging system scans

All animals were scanned at multiple time points; once prior to Cat B imaging probe injection, and then at 8, 10, and 28 h post after injection (4, 6, 24 h post upper lip formalin injection). For each scan with the IVIS Illumina Optical Imaging System (PerkinElmer), animals were anesthetized with isoflurane (induction: 5% isoflurane in 0.8 L O_2_; maintenance: 3.0% isoflurane in 0.6 L O_2_). Each imaging session lasted 5–10 min. Images were acquired with the Living Image software (PerkinElmer). The fluorescent lamp level was set at high, excitation filter to 745 nm, emission filter to indocyanine green which emits fluorescence between 750–950 nm, and each image was acquired with 20 s exposure time. Each image consisted of a photograph of the animal’s head overlayed by the detected NIRF signal. Fluorescent images were analyzed offline using Aura Imaging software (Spectral Instruments Imaging, Tucson, AZ, USA). Two rectangular regions of interest (ROI) were drawn. The signal ROI (12 mm × 18 mm) was placed over the cerebrum to measure NIRF efficiency signal. The background ROI (3 × 3 mm) was placed on an ear to measure background fluorescence efficiency. It was subtracted from the signal ROI.

### Histology

After the final IVIS Illumina Optical Imaging System scan, anesthetized animals (isoflurane 5% isoflurane in 0.8 L O_2_) were transcardially perfused with 0.1 M phosphate buffer followed by 4% paraformaldehyde in 0.1 M PBS. Brains and trigeminal ganglia were dissected and excised. Fluorescent images of the whole brain were acquired with the IVIS Illumina Optical Imaging System. Then tissue was post fixed, cryoprotected with 30% sucrose solution, embedded in tissue-plus O.C.T. compound (Fisher Scientific, Waltham, MA, USA), and stored at −80 °C.

Frozen sections of trigeminal ganglia (TG) were horizontally cut (14 μm thickness) and collected on microscopic slides. The sections were washed, blocked, and reacted simultaneously with rabbit anti-ATF3 (Santa Cruz, Dallas, TX, USA; Cat. # sc-188) and mouse anti-CGRP (Sigma-Aldrich, St. Louis, MO, USA; Cat. # C7113). Primary antibodies were visualized using goat anti-rabbit conjugated to Alexa Fluor 568 and goat anti-mouse Alexa Fluor 647. The slides were scanned using the Olympus Fluoview FV1200 laser scanning confocal microscope and software (Olympus Scientific, Waltham, MA, USA). Images were analyzed using NIH ImageJ^41^.

Coronal brain sections at bregma −2.4 to −2.92 mm (40 μm thickness) were collected in PBS. Free floating sections were washed, blocked, and reacted with rabbit anti-Iba1 (1:2000; Wako Chemicals United States, Richmond, VA, USA; Cat. # 019–19,741) or chicken anti-GFAP (Abcam, Waltham, MA, USA; Cat. # ab4674). Primary antibodies were visualized using goat anti-rabbit conjugated to Alexa Fluor 488 and goat anti-chicken Alexa Fluor 488. Images were acquired and analyzed using the Cytation 5 Image Reader (Agilent, Santa Clara, CA, USA) and Gen 5 software. The Cytation 5 was equipped with a CY7 filter cube (excitation 716/40 nm, emission 809/81 nm) to visualize the NIRF signal from the activated Cat B imaging probe.

### Statistical analysis

All data are presented as mean ± standard error of the mean. Behavioral and all fluorescent imaging data were analyzed using two-way ANOVA with Tukey or Fisher LSD post hoc testing or one-way ANOVA where appropriate using GraphPad Prism software (Dotmatics, Boston, MA, USA). A *P* value of *P* < 0.05 was considered significant.

## Results

### Optimization of in vivo imaging of extracellular Cat B

**Figure 1:**
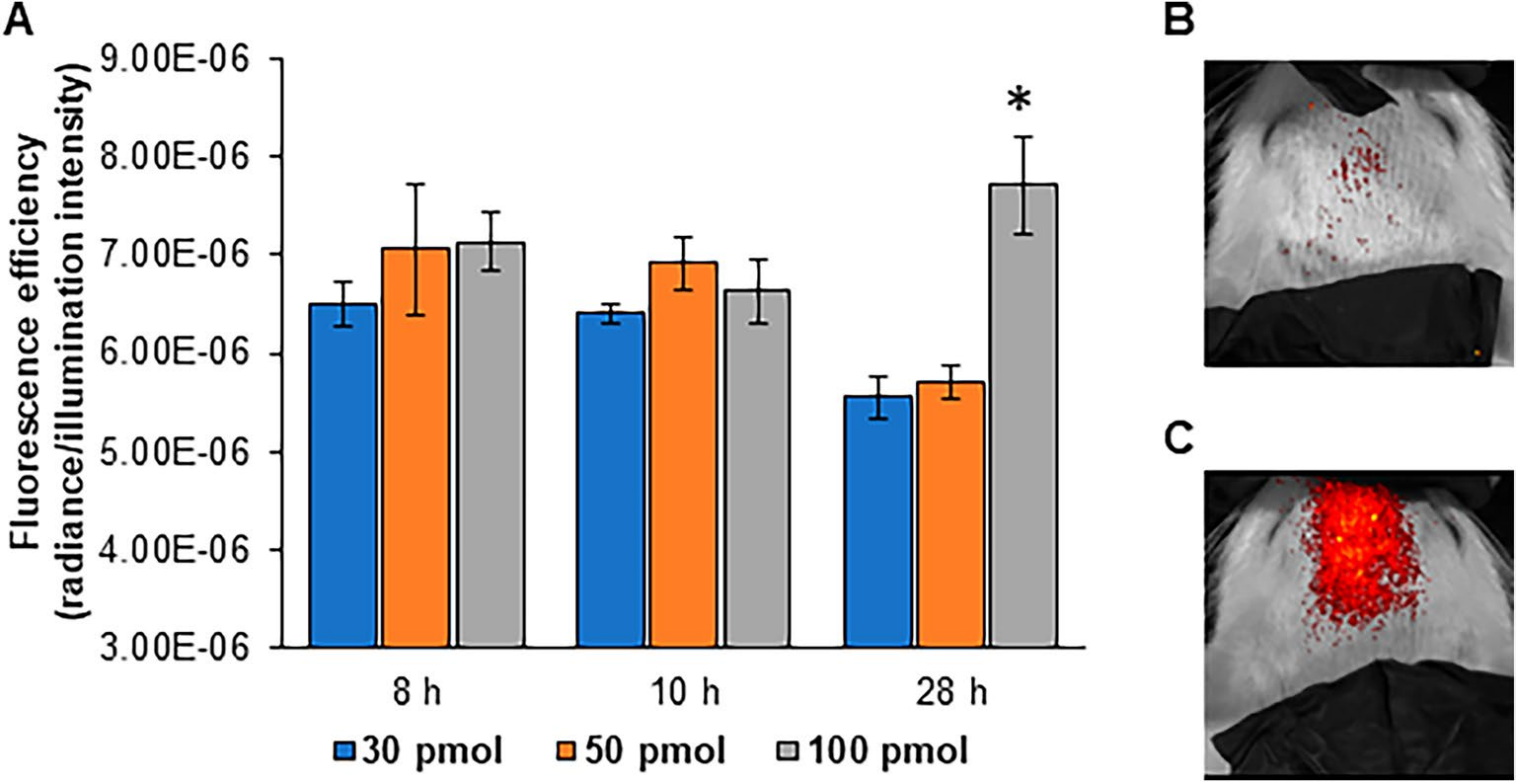
The greatest fluorescence efficiency signal (radiance/illumination intensity) was measured 28 h after intracisternal injection of the highest Cat B imaging probe concentration. (**A**) Extracellular Cat B on activated glia cleaved the specific peptide chain spacer in the imaging probe, releasing the near infrared fluorophore, and allowing it to be visualized. At 28 h post Cat B imaging probe injection, 24 h after formalin injection into the upper lip, fluorescence efficiency was highest in the 100 pmol group. * *P* < 0.05; two-way ANOVA with Tukey post hoc test. (**B**) Sample in vivo IVIS images 28 h post 30 pmol and (**C**) 100 pmol intracisternal Cat B imaging agent injection.

Three different doses of intracisternal Cat B activatable NIRF imaging probe (30, 50, 100 pmol) were tested for their efficacy to detect CNS glial cell activation in vivo up to 24 h after the orofacial formalin test. In vivo imaging of the head in prone position measured NIRF from the activated Cat B imaging probe as fluorescence efficiency (radiance/illumination intensity) using the IVIS Illumina Optical Imaging System at all time points. The highest concentration, 100 pmol, was determined to provide optimal fluorescence efficiency at 28 h post Cat B imaging probe injection (Fig. 1, two-way ANOVA with Tukey post hoc test). At 8 and 10 h post imaging probe injection, the equivalent of 4 and 6 h post orofacial formalin injection, no significant group differences were detected. The detected fluorescent signal was similar to that represented in Fig. 1B.

### Cat B imaging probe injection into the CSF did not interfere with nociceptive results of formalin study

**Figure 2:**
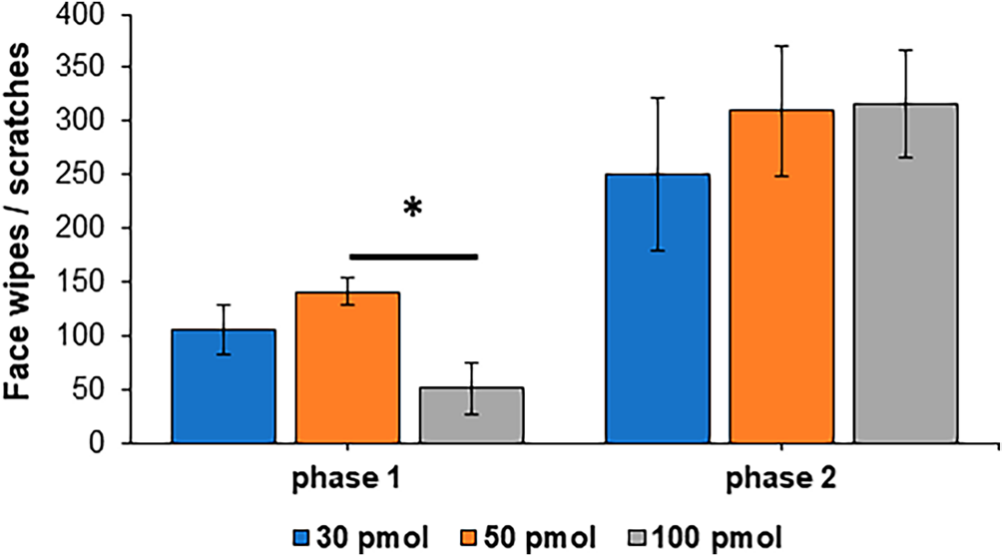
Intracisternal injection of Cat B imaging probe did not alter nociceptive behavior in phase 2 of the orofacial formalin test. Spontaneous nociceptive behavior, wiping and scratching of the face, occurred in two phases. While there was a significant difference in phase 1 with the highest dose (100 pmol), phase 2 nocifensive behavior was similar with all doses. *p* < 0.05; two-way ANOVA with Fisher LSD post hoc test.

All animals showed the typical biphasic nocifensive behavioral response after injection of formalin into the upper lip. The acute phase 1 response was separated by a quiescent 10 min period from the start of phase 2. During phase 1, the 100 pmol Cat B imaging probe group displayed significantly decreased nocifensive behavior compared to the 50 pmol group, while no behavioral differences attributable to the dilutions were detected in phase 2 (Fig. 2, two-way ANOVA with Fisher LSD post hoc test).

### Cellular distribution of Cat B imaging probe

**Figure 3:**
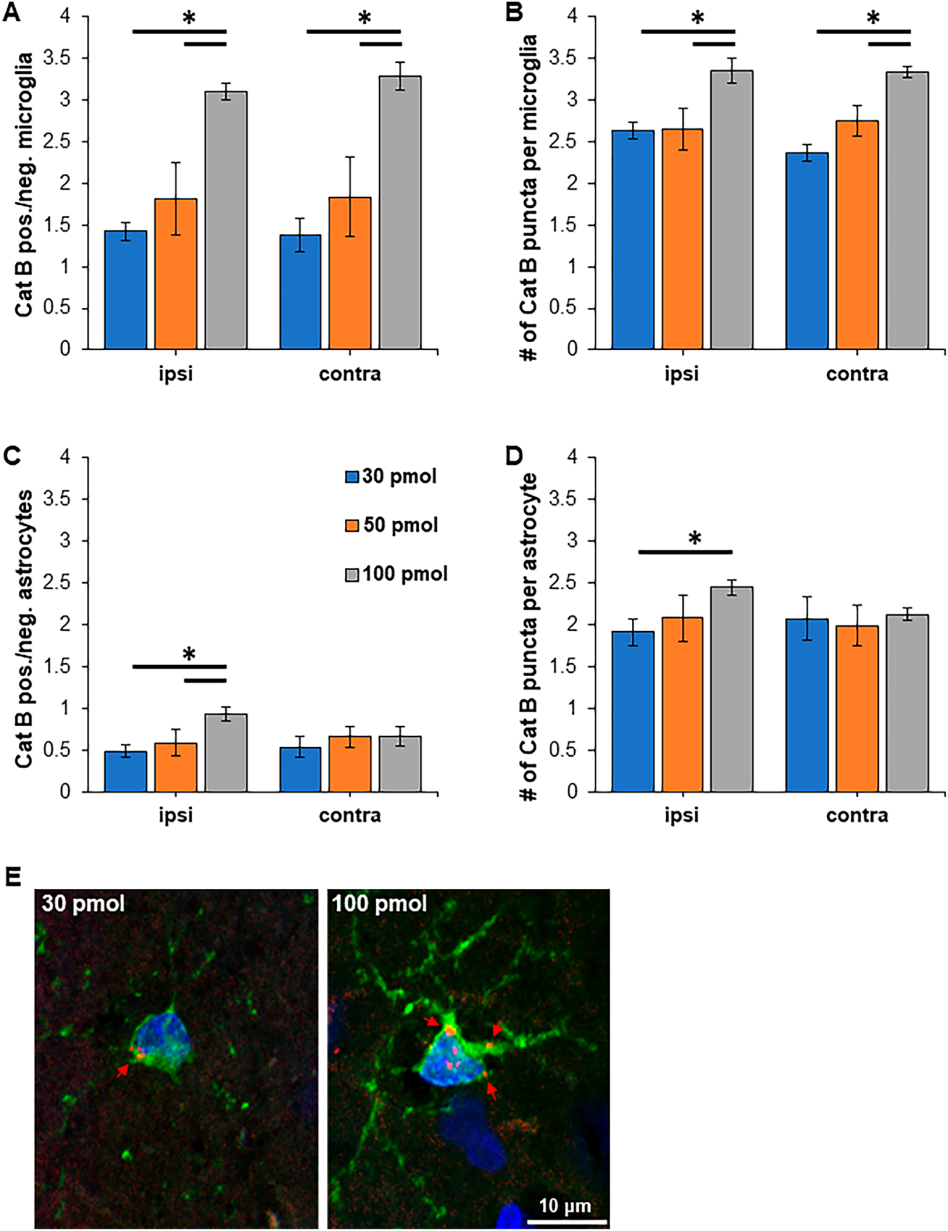
NIRF is localized on microglia and astrocytes in the somatosensory cortex concentration dependently. Immunohistochemical analysis of glial biomarkers and NIRF released from activated Cat B imaging probe were quantified in the whisker barrel somatosensory cortex in coronal sections at bregma −2.4 to −2.9 mm. (**A**) The number of NIRF labeled microglia significantly increased in a concentration dependent manner in the ipsi- and contralateral somatosensory cortex for the barrel cortex. (**B**) Similarly, the number of NIRF puncta per microglia significantly increased with higher Cat B imaging probe concentration. (**C**) The number of astrocytes with NIRF puncta and (**D**) the number of NIRF puncta increased on the ipsilateral side, while no changes were detected contralaterally. (**E**) Punctate NIRF was co-localized on Iba1 expressing microglia from the 30 pmol and 100 pmol group. * *P* < 0.05; two-way ANOVA with Tukey post hoc test. Red arrows, NIRF puncta.

**Figure 4:**
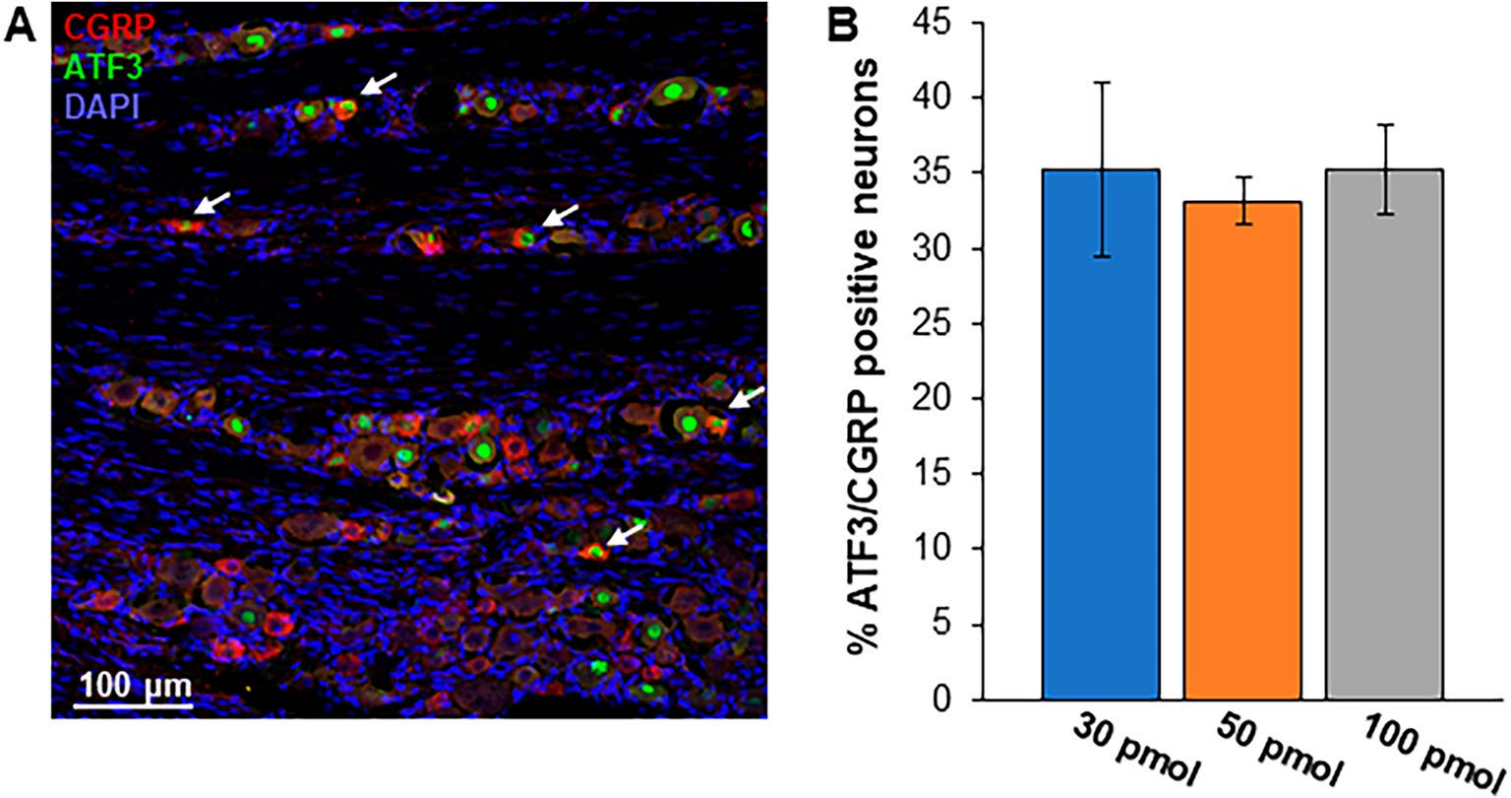
Coexpression of trigeminal ATF3 or CGRP after the formalin test was not altered by Cat B imaging probe injection. (**A**) Approximately 35% of all ATF3 positive neurons in TG also expressed CGRP. Arrow - double labeled. Red, CGRP; green, ATF3; blue, DAPI. (**B**) There was no difference between experimental groups*. P* > 0.05; one-way ANOVA.

Histological analysis of the whisker barrel somatosensory cortex in coronal sections between bregma −2.4 to −2.9 mm was conducted to determine if NIRF fluorescence could be localized on glia. On day 1 after Cat B imaging probe and formalin injections, significantly more NIRF positive microglia were detected bilaterally in the 100 pmol group compared to samples from the two lower concentrations (Fig. 3A, two-way ANOVA with Tukey post hoc test). These microglia had significantly more NIRF fluorescent puncta (Fig. 3B). Similarly, significantly more astrocytes with punctate fluorescent NIRF staining (Fig. 3C), and significantly more NIRF fluorescent puncta per astrocyte (Fig. 3D) were detected in the ipsilateral whisker barrel somatosensory cortex of the 100 pmol group indicative of increased extracellular Cat B. However, on the side contralateral to the formalin injection, no differences were detected (Fig. 3C,D). Figure 3E shows a microglia from the 30 and 100 pmol groups with punctate NIRF fluorescence. No activated Cat B imaging probe fluorescence was detected in the TG (data not shown). Cat B imaging probe injection did not alter immunohistochemical staining of TG for CGRP expressed by peptidergic nociceptors nor transcription factor ATF3, a biomarker for injured neurons. Approximately 35% of all ATF3 positive sensory neurons also expressed CGRP (Fig. 4). No group differences were detected (one-way ANOVA).

## Discussion

Glial activation after peripheral inflammation/injury is well documented. The peripheral insult causes hyperactivity in nociceptors, resulting in excess release of neuropeptides and other pro-inflammatory signaling molecules into the CNS. Ramified microglia, innate immune cells of CNS, constantly scan and monitor the environment. During nociceptor hyperactivity, they are activated by released endogenous damage-associated molecular patterns (DAMPs) such as cyclic nucleotides, neuropeptides, and other pro-inflammatory mediators^18,42^. This can activate microglial purinergic P2X7 receptors, causing lysosomal leakage and increased cytoplasmic Cat B protease which primes NLRP3, resulting in activation of inflammasomes, and interleukin 1β release^25,43,44^. Changes in subcellular localization of Cat B are critical for its multitude of functions. Typically localized in acidic lysosomes, this cysteine lysosomal protease acts as an exopeptidase and participates in autophagy, protein degradation and recycling autophagy^23,45^. When secreted into pH neutral environments, Cat B functions as an endopeptidase. It can modify other cytoplasmic and extracellular signaling molecules as well as the extracellular matrix^15,20,27,28,46,47^.

### Subcellular localization of activated Cat B imaging probe NIRF

Extracellular Cat B activity was detected in vivo by using an injected Cat B specific protease-activatable NIRF imaging probe in the IVIS. Histologically, increased extracellular Cat B was evidenced by more NIRF puncta on microglia and astrocytes. Optical imaging stacks taken with the confocal microscope (0.5 μm z-steps) showed a close association of NIRF from the activated Cat B imaging probe with the cell membrane and not in cytoplasmic organelles. To date, injectable protease-activated NIRF probes are designed for the detection of membrane-bound or soluble extracellular proteins^29,48^. Multiple studies have shown that Cat B is secreted and can be associated with the cell membrane in specialized microdomains such as lipid rafts or caveolae to modulate cell adhesion and receptor molecules or degrade the extracellular membrane^28,49–51^. Cat B is a lysosomal cysteine protease. It is possible that the punctate NIRF signal originated from microdomains in the cell membrane as well as endocytosed Cat B containing vesicles that became part of the endosomal and lysosomal intracellular compartments^52^.

### Central but not peripheral involvement of Cat B in inflammatory pain

The CSF flows from the subarachnoid spaces through sensory ganglia along brain and peripheral nerves^53^. The finding of activated Cat B probe associated with glial cells in the brain but not in TG point to the role of extracellular Cat B in central but not peripheral aspects of inflammatory pain. This finding is supported by previous observations in experimental inflammatory pain using intraplantar complete Freund’s adjuvant (CFA) injections in a Cat B knock-out mouse model^54^. Specifically, the authors found that Cat B deficiency or its inhibition did not alter peripheral inflammation, however, spinal microglia did not express or secrete mature IL-1β and IL-18. This indicates that in the periphery, inflammatory cytokines and chemokines mediate pain in a Cat B-independent fashion while centrally, hyperalgesia is dependent on glial Cat B with inflammatory mediators conveying peripheral inflammation to the CNS^7,15^.

### Technical considerations

Each molecule of the compound under study consists of Cat B protease specific peptide chain spacer that connects two NIRF dye molecules. The fluorescence is quenched by their close proximity, and only upon enzymatic cleavage of the peptide chain spacer are the fluorophores released and thus activated^31,48^. Due to its large size (23 kDa), it is unable to penetrate the intact blood–brain barrier^55^ and thus applied directly into the CSF with intracisternal injections. All animals survived the injections. Although there were group differences for nocifensive behavior in phase 1 of the orofacial formalin test, none were determined during phase 2. Interactions between intracisternal injection and isoflurane may have caused behavioral variability in the early phase of this inflammatory test that did not have an effect on the late phase^56^.

It was determined that a 400-fold lower Cat B imaging probe concentration than recommended for intravenous application was needed for optimal fluorescence in our study. In vivo IVIS imaging of the head provided two dimensional pictures of NIRF. The doses tested were within the rising aspect of the dose–response curve. Optimal activated Cat B imaging probe fluorescence is usually 8–12 h after intravenous injection^55^. However, we noted a signal increase from the 12 to 28h measurements for 100 pmol concentration, which may have been caused by slower fluorophore degradation in the CNS and/or slower fluid distribution in the closed CSF system.

Transcardial perfusion with formaldehyde fixed the released fluorophore from the activated Cat B imaging probe in the tissue, allowing its visualization in tissue sections. We identified punctate NIRF staining on microglia as well as astrocytes 24 h after the inflammatory insult. NIRF intensity was dose dependent comparable to in vivo fluorescence. Microglia had significantly more punctate NIRF staining than astrocytes similar to previous reports that inflammation-induced Cat B expression in microglia is significantly greater compared to other inflammatory cells^7,15,57,58^. Pro-inflammatory microglia activate astrocytes, which then increase DAMP signaling^7^. In the present study, we focused on the primary sensory cortex due to limited depth resolution and two-dimensional imaging, limitations in in vivo IVIS. Bilateral concentration dependent differences of punctate microglia staining were not seen with astrocytes. The contralateral staining differences in astrocytes cannot readily be explained and will require additional study.

It is known that inflammatory stimuli activate the CNS pain circuitry. However, as a limitation of the present method, many of the brain regions involved are located deep in the skull, making them inaccessible for this type of in vivo imaging by IVIS. Three-dimensional time course studies are needed to further investigate the role of Cat B in the CNS in inflammatory pain models.

### Advantages of using a protease-specific activated NIRF probe to image activated glia

The present method allows for repeated in vivo imaging of the same animal after a single injection of the protease activated NIRF probe. This allows for the establishment of time-course series with a fraction of the animals that ex vivo whole organ imaging or histological analyses would require. It can used in wildtype animals without the need for genetic manipulation. The protease activated NIRF probe has the advantage of visualizing activated glia cells that express extracellular Cat B. There are other protease activated NIRF probes commercially available, allowing for the study of other cell types. The NIRF compound is non-toxic and excreted within several days (according to manufacturer), allowing for repeated injection and in vivo imaging. There are presently 2 established methods for glial imaging in vivo in animal models. An increase of glial cells can be measured in the CNS without the need of a probe using T2-weighted MRI images. Otherwise, a radioactive tracer can be used to label glia prior to PET imaging^59^. Both methods are financially prohibitive and not available in most animal facilities. Use of protease-cleavable compounds with NIR fluorophores are extremely beneficial for these types of investigations.

## Conclusion

The data here demonstrate a time- and concentration-dependent efficacy of extracellular Cat B visualization in activated glia after orofacial formalin was injected. The NIRF probe did not alter known protein expression changes in this inflammatory pain model nor did it affect the nocifensive behavior. Intracisternal injection of this compound is well suited for future studies of extracellular CNS proteinases when the blood–brain barrier is not disrupted.

## Data Availability

All data generated or analyzed during this study are included in this published article.

## Abbreviations

Cat B: Cathepsin B
CFA: Complete Freund’s adjuvant
CGRP: Calcitonin gene-related peptide
CNS: Central nervous system
CSF: Cerebrospinal fluid
DAMP: Damage-associated molecular pattern
FAST: Fluorescent activatable sensor technology
GFAP: Glial fibrillary acidic protein
IVIS: In Vivo Imaging System
NIRF: Near-infrared fluorescence
PBS: Phosphate-buffered saline
ROI: Region of interest
TG: Trigeminal ganglion
